# Identification and Analysis of Stress-Associated Protein (SAP) Transcription Factor Family Members in *Pinus massoniana*

**DOI:** 10.3390/plants14111592

**Published:** 2025-05-23

**Authors:** Yulu Zhao, Xingyue Ren, Jingjing Zhang, Wenya Yu, Qiong Yu, Kongshu Ji

**Affiliations:** 1 State Key Laboratory of Tree Genetics and Breeding, Nanjing Forestry University, Nanjing 210037, China; 2 Co-Innovation Center for Sustainable Forestry in Southern China, Nanjing Forestry University, Nanjing 210037, China; 3 Beijing National Laboratory for Molecular Sciences, Beijing 100190, China

**Keywords:** stress-associated proteins (SAPs), *Pinus massoniana*, expression pattern, abiotic stress

## Abstract

Stress-associated proteins (SAPs), belonging to the A20/AN1 zinc finger protein family, are key regulators in plant stress responses. Despite their importance, studies on the SAP gene family in *Pinus massoniana* are still relatively scarce. This study aimed to systematically identify and characterize SAP genes in *P. massoniana* and to explore their potential roles in stress response mechanisms. A total of 17 *PmSAP* genes were identified from *P. massoniana*. Phylogenetic analysis revealed that these genes group into five distinct clades, and 10 conserved motifs were identified. Using transcriptome data and qRT-PCR, we analyzed their expression patterns and employed yeast systems to validate their transcriptional activities. The responses of *PmSAP* gene family members to different stress treatments showed significant differences. For example, *PmSAP8* and *PmSAP12* responded strongly to ABA, MeJA, and H_2_O_2_ treatments, while *PmSAP3* and *PmSAP5* showed significant upregulation under ETH and NaCl stress. Yeast experiments indicated that *PmSAP6/8/12* were transcriptional activators, and *PmSAP3* and *PmSAP5* were transcriptional suppressors. The identification and preliminary analysis of *PmSAP* genes provided a theoretical foundation for understanding stress resistance mechanisms in *P. massoniana.*

## 1. Introduction

The growth and development of plants are significantly affected by diverse biotic and abiotic stress conditions. Among the key regulatory components in plants, transcription factors (TFs) function by binding to particular DNA sequences—known as cis-regulatory elements—located in the promoter regions of target genes. During various growth stages, transcription factors serve as vital regulators of both developmental progression and abiotic stress, such as drought, salt stress, and low and high temperature [1,2]. Recent research has confirmed the function of multiple critical TFs in stress responses, making them appropriate targets for improving stress resistance across numerous plant species. These include mitogen-activated protein kinases (MAPKs), DRE/CRT-binding transcription factors (DREBs/CBFs), NAC domain-containing transcription factors, AP2/ERF transcription factors, stress-associated proteins (SAPs), heat shock factors/proteins (HSFs/HSPs), high-affinity K^+^ transporters, and regulatory components of abscisic acid (ABA) receptors [2,3,4,5,6,7,8]. Among these factors, SAPs constitute a specialized group of zinc finger proteins (ZFPs) known to mediate abiotic stress adaptation in plants and additionally participate in human immune regulation [1,9,10].

Members of the SAP protein family contain either A20 or AN1 zinc finger domains, or both. Originally discovered in human umbilical vein endothelial cells as part of a TNF-α-inducible protein, the A20 domain exhibits a conserved Cx_2–4_Cx_11_Cx_2_C sequence motif [11]. The AN1 domain was recognized initially as a potential zinc finger domain in the protein encoded by maternal RNA from the animal hemisphere 1 (AN1) of *Xenopus laevis* [12]. This domain is frequently associated with the A20 zinc finger, and this type of protein is present in all eukaryotes [13]. Accumulating evidence indicates that numerous SAP genes participate in plant responses to abiotic stress. For example, *AtSAP5* overexpressing plants significantly improved drought resistance relative to wild-type (WT) controls [14,15]. *AtSAP13* overexpression confers improved tolerance to salinity, drought, and heavy metals [16]. *OsSAP7* overexpressing rice shows reduced sensitivity to drought, cold, and salt stress [17]. *MdSAP15* overexpression enhances osmotic stress tolerance in *Arabidopsis* seedlings [18]. However, not all SAPs positively regulate stress responses. Under ABA treatment, *AtSAP9* mutants display higher germination rates than WT, whereas *AtSAP9* overexpressing plants exhibit suppressed germination [19], suggesting a negative regulatory role in certain stress conditions.

Furthermore, emerging evidence demonstrates that SAPs also play significant roles in plant defense against biotic stressors. Notably, ectopic expression of *OsSAP1* in transgenic tobacco plants conferred enhanced resistance to bacterial pathogens [20]. Transgenic plants overexpressing either *AtSAP5* or its ortholog in *P. aphrodite*, *Pha13*, displayed enhanced viral resistance, while *AtSAP5*-RNAi lines exhibited greater susceptibility to viral infection compared to wild-type controls [21]. When *SlSAP3* expression was inhibited in tomato (*Solanum lycopersicum*), the plants showed diminished resistance to Pst (*Pseudomonas syringae* pv. tomato) DC3000 infection, whereas *SlSAP3* overexpression enhanced pathogen resistance [22].

Up to the present moment, systematic identification and characterization of SAP family genes have been conducted in several plant species; for example, rice (*Oryza sativa*) [23], tomato [24], cotton (*Gossypium hirsutum*) [25], *Medicago truncatula* [26], *Brassica napus* [27], *Arabidopsis* and soybean [28], cucumber (*Cucumis sativus*) [29], sweetpotato (*Ipomoea batatas*) [30], sunflower (*Helianthus annuus*) [31], potato (*Solanum tuberosum* L.) [32], *Tamarix hispida* [33], grapevine (*Vitis vinifera* L.) [34], castor bean (*Ricinus communis*) [35], wheat (*Triticum aestivum* L.) [36], and poplar 84K (*Populus alba* × *P. tremula var. glandulosa*) [37].

*Pinus massoniana* Lamb. is a widely distributed coniferous species in China and represents one of the nation’s most economically important timber trees. Its derivatives, including pine resin, pine pollen, and pine needles, serve as valuable raw materials with significant commercial value. However, increasing industrialization has led to environmental deterioration, exposing *P. massoniana* to various adverse conditions where both biotic and abiotic stresses cause substantial economic losses [38]. Consequently, enhancing stress resistance in *P. massoniana* has become a research priority.

Despite that the SAP family members are crucial for plant growth and stress resistance, systematic research has not yet been investigated in *P. massoniana*. Therefore, the general objective of this study is to identify and comprehensively characterize the SAP gene family in *P. massoniana* and to explore their potential roles in abiotic stress responses through a combination of bioinformatics and stress response expression profile analysis. To achieve this, we identified *PmSAP* genes from the *P. massoniana* transcriptome and analyzed their physicochemical properties, phylogenetic analysis, conserved motifs, and domain features. In addition, we also investigated the expression patterns of *PmSAP* in diverse tissues when exposed to various abiotic conditions and their responses to various phytohormone treatments. Drawing upon expression data from RNA-seq and qRT-PCR experiments, several genes were chosen for subsequent functional analysis. These outcomes not only provide fundamental insights for studying the SAP transcription factor family members in *P. massoniana* but also provide theoretical research for studying the response mechanism of *P. massoniana* to external stress.

## 2. Results

### 2.1. Identification of SAP Family Proteins in P. massoniana

In order to identify SAP genes, we implemented the Hidden Markov Model (HMM) (PF01428, PF01754) to search four *P. massoniana* transcriptomes databases. After eliminating sequences lacking conserved domains and redundant entries, we detected 17 SAP proteins in the *P. massoniana* transcriptome. The protein sequences of these 17 *PmSAP* TFs, which were designated *PmSAP1* through *PmSAP17* (Table S1), were utilized to analyze their physicochemical properties. These protein sequences varied in length from 63 amino acids (aa) in *PmSAP11* to 334 amino acids (aa) in *PmSAP4*. The range of molecular weights (MW) was 7.08 kD for *PmSAP11* and 36.86 kD for *PmSAP4*. The range of values for the isoelectric point (pI) was 7.47 (*PmSAP6*) to 9.65 (*PmSAP7*). The aliphatic amino acid index values demonstrated a range of 46.29 (*PmSAP16*) to 72.78 (*PmSAP13*). The range of the instability index (II) is from 25.73 (*PmSAP1*) to 62.19 (*PmSAP16*). The grand average of hydropathicity (GRAVY) is all negative, confirming that *PmSAP*s are hydrophilic proteins (Table S2).

### 2.2. Phylogenetic Analysis of the SAP Proteins

**Figure 1:**
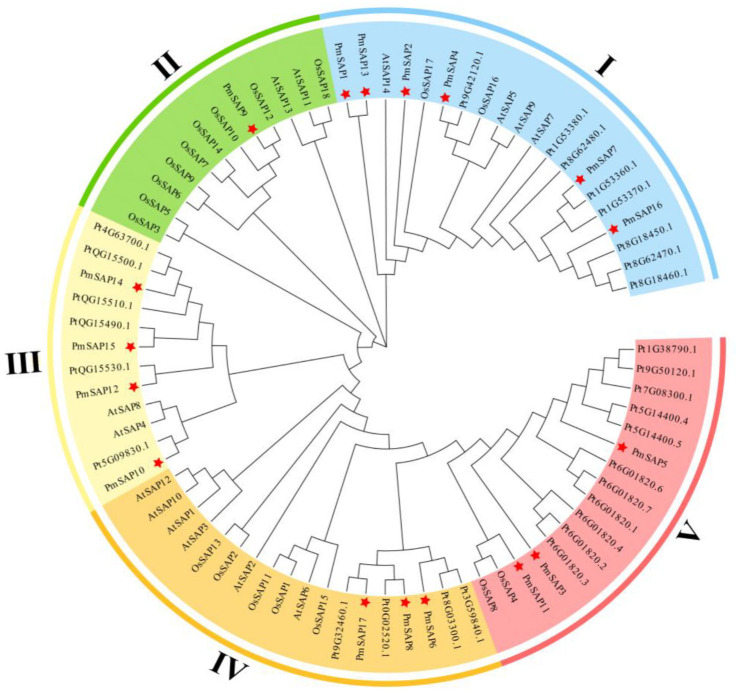
Phylogenetic tree of the SAP gene family. Pm: *Pinus massoniana*; At: *Arabidopsis thaliana*; Pt: *Pinus tabuliformis*; Os: *Oryza sativa*. Different colored rings represent different clades, and the red stars indicate the SAP proteins of *P. massoniana*.

Based on 17 SAP protein sequences of *P. massoniana*, 14 SAP protein sequences of *Arabidopsis thaliana*, 29 SAP protein sequences of *Pinus tabuliformis*, and 18 SAP protein sequences of *Oryza sativa*, we constructed a phylogenetic tree using MEGA7, employing the neighbor-joining (NJ) algorithm with 1000 bootstrap replicates (Figure 1). All examined SAPs were classified into five distinct clades (Clades I–V). Among them, Clade I (the largest subgroup) comprised twenty members, including six *PmSAP* genes (*PmSAP1*, *PmSAP2*, *PmSAP4*, *PmSA7*, *PmSAP13*, and *PmSAP16*), representing 35% of the total SAP family *P. massoniana*; Clade II contained the most limited representation, with only one *PmSAP* gene (*PmSAP9*) identified; Clade III included four *PmSAP* genes (*PmSAP10*, *PmSA12*, *PmSAP14*, and *PmSAP15*), showing intermediate conservation; Clades IV and V each contained three *PmSAP* genes; and Clade IV contained *PmSA6*, *PmSAP8*, and *PmSAP17*, while Clade V included *PmSA3*, *PmSAP5*, and *PmSAP11*. All clades contained *P. massoniana* SAP family members. However, only Clade I and Clade IV included SAP members from four species. This might be due to the different evolutionary patterns of different types of plants, resulting in the loss of some lineage-specific genes.

### 2.3. Analysis of Motifs and Domains in PmSAP Family Proteins

**Figure 2:**
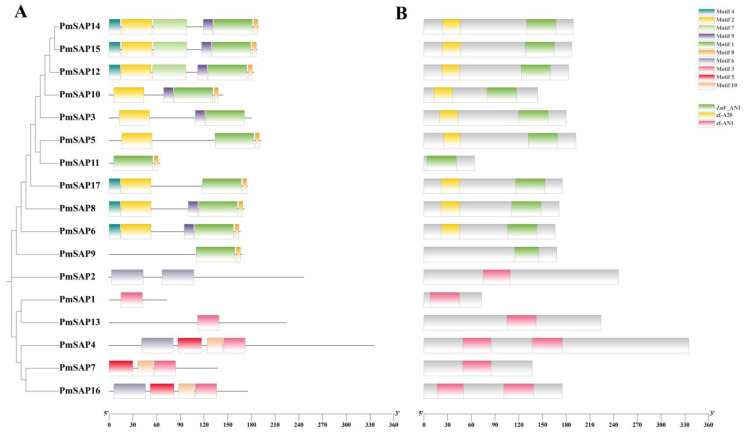
Analysis of the distribution of motifs and domains of SAP proteins in *P. massoniana*. (**A**) Distribution of PmSAP protein motifs. 10 different motifs (motif 1–10) are represented by different colors. (**B**) Distribution of PmSAP protein domains. Green represents the ZnF-AN1 domain, yellow represents the zf-A20 domain, and red represents the zf-AN1 domain.

Through the MEME website, we identified 10 conserved motifs among the 17 *PmSAP* TF families, and they were named Motifs 1–10. Notably, Motif 1, Motif 2, and Motif 3 represent characteristic domains of SAP TFs (Figure 2A). The 10 motifs exhibit amino acid lengths from 6 to 50 (Table S3). The analysis of conserved domains showed that every *PmSAP* protein possesses the AN1/A20 domain, indicating that the structure of *PmSAP* TFs is complete. Among them, *PmSAP3*, *PmSAP5*, *PmSAP6*, *PmSAP8*, *PmSAP10*, *PmSAP12*, *PmSAP14*, *PmSAP15*, and *PmSAP17* contain the A20 domain and the AN1 domain; *PmSAP1*, *PmSAP2*, *PmSAP7*, *PmSAP11*, and *PmSAP13* have a single AN1 domain; and *PmSAP4* and *PmSAP16* each have two AN1 domains (Figure 2B). Furthermore, the distribution and characteristics of reverse motifs within the same SAP clade family are similar to those of the classification results of the phylogenetic tree. Such patterns demonstrate both functional similarity and evolutionary conservation among genes within the same phylogenetic clades.

### 2.4. Transcription Profile Analysis of PmSAP Genes During Drought Treatment

**Figure 3:**
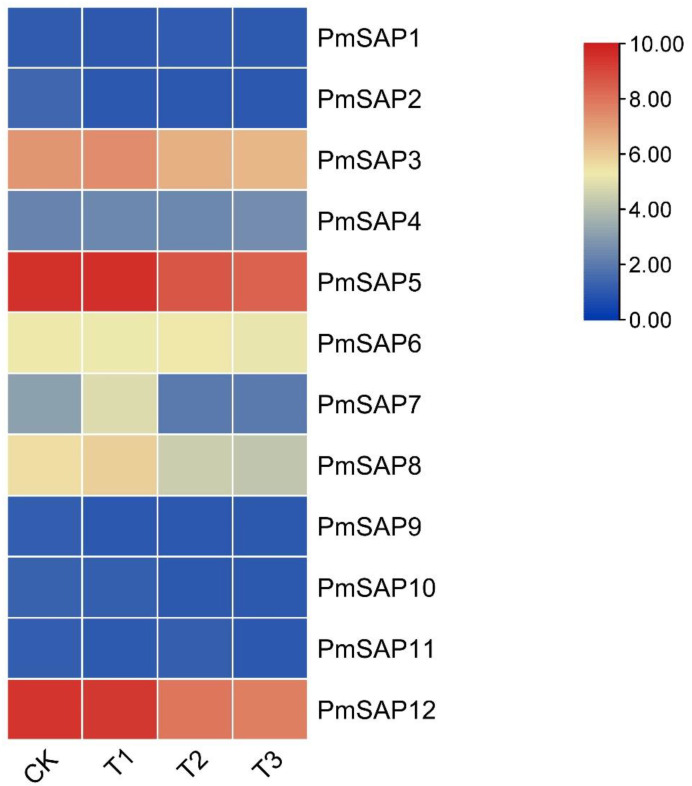
Analysis of the transcriptional profiles of *PmSAP* genes of *P. massoniana* during three different types of drought stress conditions. CK: control group, T1: mild drought condition, T2: moderate drought condition, T3: severe drought condition. A heatmap was constructed by normalizing along rows based on the log_2_ (FPKM + 1) values. The gene expression level is depicted by color intensity, scaling from high (red) to low (blue).

In order to investigate the mechanism of action of the *PmSAP* genes during drought stress conditions, we constructed a heatmap of the *PmSAP* gene expression using the drought stress transcriptome data (PRJNA595650). Due to the fact that the expression levels of some *PmSAP* TF genes are lower than the detectable values, we analyzed the expression profiles of *PmSAP1*-*PmSAP12* genes under drought conditions (Figure 3). Our analysis revealed distinct drought-responsive expression profiles among *PmSAP* genes. For instance, compared with the control group, we observed similar transcriptional responses among *PmSAP3*, *PmSAP5*, *PmSAP7*, and *PmSAP8* genes. While showing modest upregulation during mild drought, these genes exhibited downregulation under both moderate and severe stress conditions. The expression level of the *PmSAP12* gene continuously decreased under drought response. These findings indicate that most *PmSAP* genes are responsive to drought treatment and may play a significant role in drought stress adaptation. The differential expression patterns suggest functional diversification among *PmSAP* family members in response to varying drought intensities.

### 2.5. Subcellular Localization of PmSAP TFs

**Figure 4:**
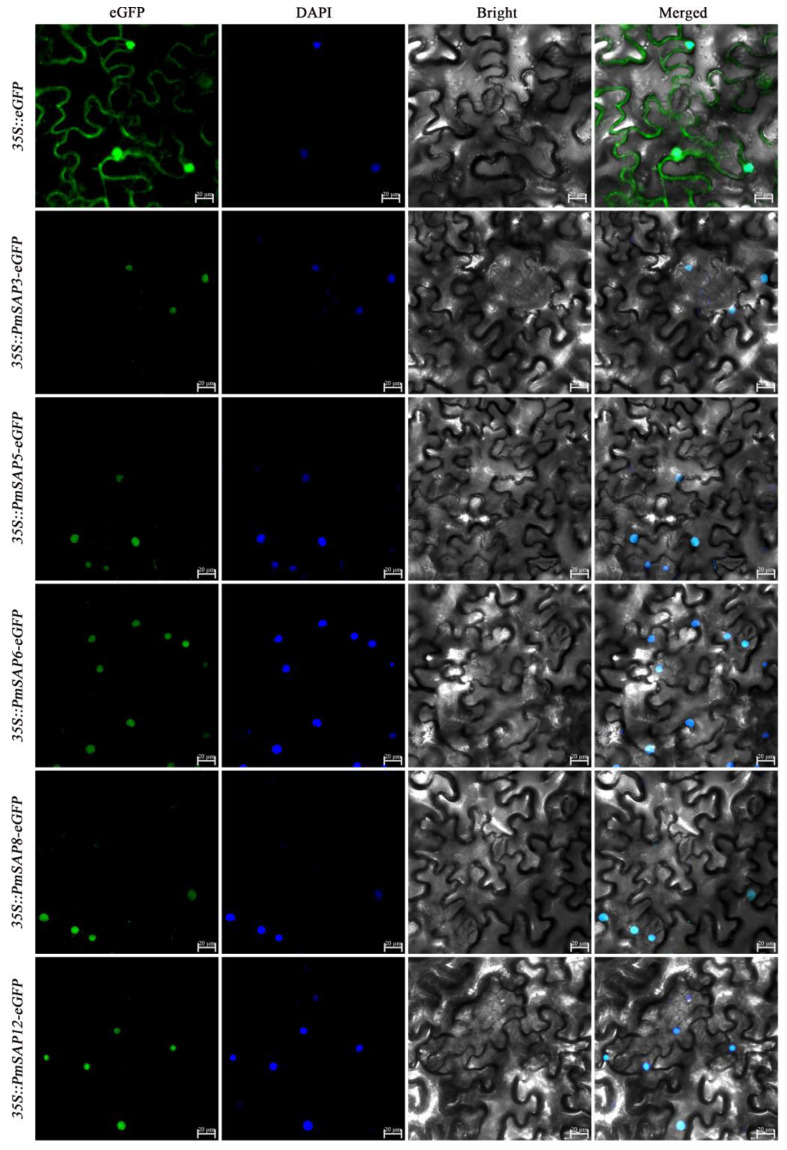
Subcellular localization of PmSAP proteins. Transient expression of 35S::eGFP (control) and 35S::*PmSAP3/5/6/8/12*-eGFP in tobacco leaves. The scale bar is 20 µm.

Subcellular localization prediction using Cell-PLoc 2.0 indicates that all *PmSAP* TFs are nuclear localization proteins (Table S4). In order to further verify whether the subcellular localization of *PmSAP* TFs is nuclear localization, we selected five highly expressed genes (*PmSAP3*, *PmSAP5*, *PmSAP6*, *PmSAP8*, and *PmSAP12*) from the drought stress transcriptome for transient transformation experiments. Fluorescence signals were observed in transiently transformed tobacco leaves (Figure 4). It was found that the GFP signal was distributed in the cells of the control group. In contrast, the GFP fluorescence signals in *PmSAP3*, *PmSAP5*, *PmSAP6*, *PmSAP8*, and *PmSAP12* are only displayed within the cell nuclei. These findings demonstrate that PmSAP proteins are localized in the nucleus, which is consistent with the predicted results.

### 2.6. Expression Patterns of PmSAP Genes in Various Tissues

**Figure 5:**
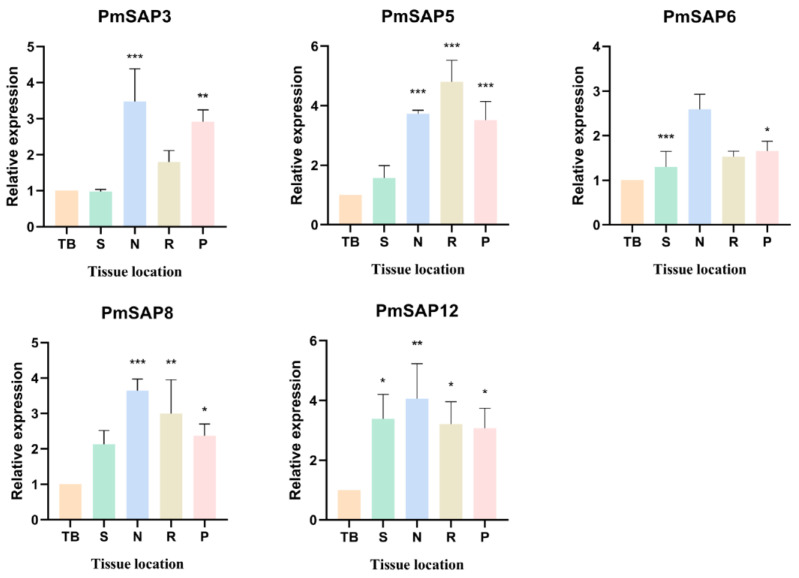
Expression levels of *PmSAP3/5/6/8/12* genes in five distinct tissues of *P. massoniana*. TB: terminal bud, S: stem, N: needle, R: root, P: phloem. The expression levels are presented as mean ± standard deviation (SD) derived from three independent biological replicates (each with three technical replicates). The asterisk (*) denotes statistically significant differences in expression levels relative to controls (* *p* < 0.05, ** *p* < 0.01, *** *p* < 0.001).

The expression levels of five selected *PmSAP* genes (*PmSAP3*, *PmSAP5*, *PmSAP6*, *PmSAP8*, and *PmSAP12*) in five tissues (terminal bud (TB), stem (S), needle (N), root (R), and phloem (P)) were analyzed by quantitative reverse transcription PCR (qRT-PCR). These genes were selected based on their high expression levels in the drought stress transcriptome. The results (Figure 5) showed that the expression level of *PmSAP3* was significantly higher in needles and phloem compared to other tissues, with expression levels approximately 3.5 times and 3 times relative to the terminal bud. The expression level of *PmSAP5* was approximately 3.7, 4.8, and 3.5 times that of the terminal bud in the needle, root, and phloem, respectively. Needle tissues exhibited relatively high *PmSAP6* expression, measuring 2.6 times the level detected in the terminal bud, while the expression levels in the stem, root, and phloem were relatively consistent with that of the terminal bud. The expression level of *PmSAP8* was the highest in the needles, being approximately 3.6 times the terminal bud levels, while the stem, root, and phloem showed 2.1, 3.0, and 2.4 times differences, respectively. Relative to the terminal bud, *PmSAP12* expression was upregulated 3.4 times in the stem, 4.1 times in the needle, 3.2 times in the root, and 3.1 times in the phloem. In conclusion, *PmSAP5* was predominantly expressed in roots, whereas other *PmSAP* transcription factors exhibited maximal expression levels in needles.

### 2.7. Expression Pattern of PmSAPs Under Different Treatments

**Figure 6:**
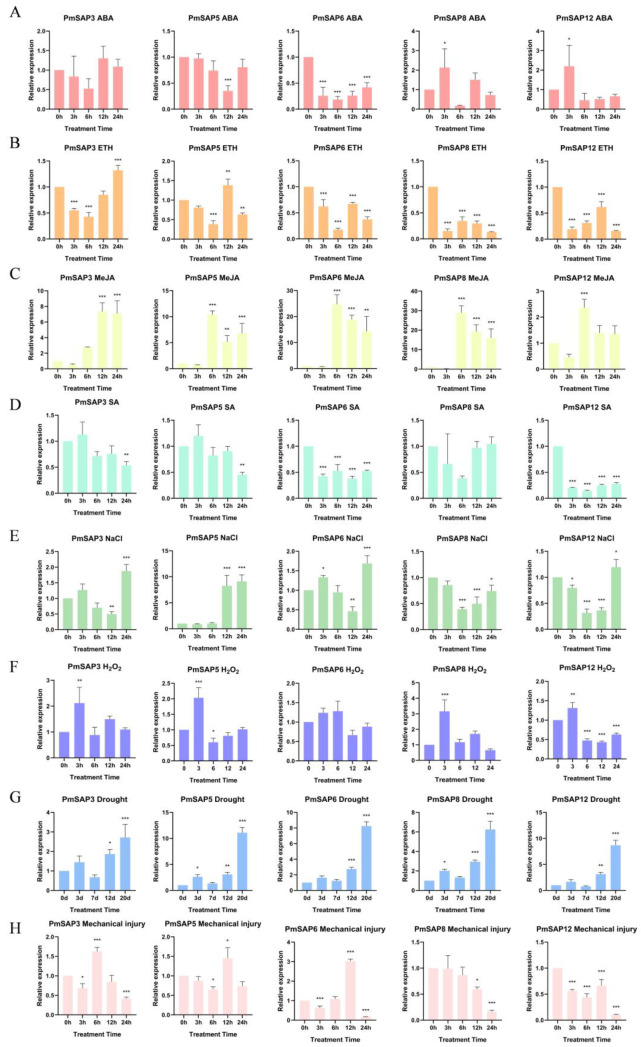
Expression patterns of *PmSAP3/5/6/8/12* genes under different treatments. (**A**) ABA, (**B**) ETH, (**C**) MeJA, (**D**) SA, (**E**) NaCl, (**F**) H_2_O_2_, (**G**) drought, (**H**) mechanical injury. The asterisk (*) denotes statistically significant differences in expression levels relative to controls (* *p* < 0.05, ** *p* < 0.01, *** *p* < 0.001).

We analyzed the expression patterns of *PmSAP* genes under different treatments (Figure 6). Under ABA treatment, *PmSAP8* and *PmSAP12* showed obvious positive responses, both reaching their maximum expression levels at 3 h and having the lowest expression levels at 6 h. The expression level of *PmSAP6* did not change much in response to ABA treatment; *PmSAP3* expression increased progressively, peaking at 12 h, whereas *PmSAP5* levels declined to a minimum at the same timepoint. There were no significant differences in *PmSAP3* throughout the process (Figure 6A). Under the treatment of ETH, the expression levels of *PmSAP6*, *PmSAP8*, and *PmSAP12* were all downregulated and inhibited throughout the treatment. *PmSAP3* showed significant downregulation at 3 h and 6 h, followed by upregulation at 12 h, and a significant upregulation at 24 h, reaching the maximum expression level. *PmSAP5* reached the maximum expression level at 12 h and gradually decreased at 24 h, showing a trend of decreasing, increasing, and then decreasing (Figure 6B). Under MeJA treatment, all the genes showed significant responses to MeJA. Among them, *PmSAP5*, *PmSAP6*, *PmSAP8*, and *PmSAP12* showed significant upregulation in expression at 6 h and reached the maximum value. Subsequently, the expressions of *PmSAP6*, *PmSAP8*, and *PmSAP12* gradually decreased, while the expression of *PmSAP5* significantly decreased at 12 h and slightly increased at 24 h. *PmSAP3* demonstrated significantly increased expression at the 12 h and 24 h intervals (Figure 6C). Under SA treatment, both *PmSAP3* and *PmSAP5* exhibited a similar expression pattern: their expression levels initially increased at 3 h post-treatment, followed by a significant decline at 24 h. Meanwhile, *PmSAP6* and *PmSAP8* demonstrated significantly decreased and inhibited expression levels throughout the treatment (Figure 6D). Under NaCl stress conditions, *PmSAP3*, *PmSAP6*, and *PmSAP12* exhibited similar expression patterns. All three showed a slight increase at 3 h and then decreased at 6 h and 12 h, reaching the maximum expression level at 24 h. *PmSAP5* showed no significant change in expression at 3 h and 6 h but had a significant response at 12 h and 24 h and reached the maximum peak at 24 h, which was approximately 9 times that of 0 h. *PmSAP8* showed no significant response to NaCl (Figure 6E). Under the H_2_O_2_ treatment, the expression levels of all genes except *PmSAP6* significantly increased, and reached the maximum expression level at the 3-h time point. Among them, *PmSAP8* had the highest expression, approximately 3.2 times that of the untreated condition (0 h). However, *PmSAP6* showed no significant expression changes (Figure 6F). Under drought treatment, all the genes exhibited significant responses to drought stress, and their expression patterns were similar. After 3 days of drought treatment, *PmSAP* expression levels increased and decreased after 7 days. Then, they significantly increased after 12 days of treatment, and reached the peak at 20 days, which was approximately 1.9 times, 11 times, 8.2 times, 6.3 times, and 8.7 times relative to day 0 (Figure 6G). After mechanical injury, *PmSAP8* and *PmSAP12* expression levels decreased and reached the lowest values at 24 h, indicating that the expressions of *PmSAP8* and *PmSAP12* were gradually inhibited after mechanical injury. The expressions of *PmSAP5* and *PmSAP6* were downregulated at 3 h and significantly upregulated at 12 h and reached the highest expression level. *PmSAP3* exhibited an expression pattern, with an initial downregulation followed by an upregulation and subsequent decline (Figure 6H). The above results indicate that *PmSAP* family members exhibited markedly divergent responses across various stress conditions. Among them, *PmSAP8* and *PmSAP12* respond strongly to ABA, MeJA, and H_2_O_2_ treatments; *PmSAP3* and *PmSAP5* show significant upregulation under ETH and NaCl stress, while drought stress can induce sustained high expression of all *PmSAP* genes. These differential expression characteristics suggest that different *PmSAP* genes potentially contribute unique functions to plant stress response systems, providing important clues for in-depth analysis of their molecular mechanisms.

### 2.8. Transcriptional Activity Analysis of PmSAP3, PmSAP5, PmSAP6, PmSAP8, and PmSAP12

**Figure 7:**
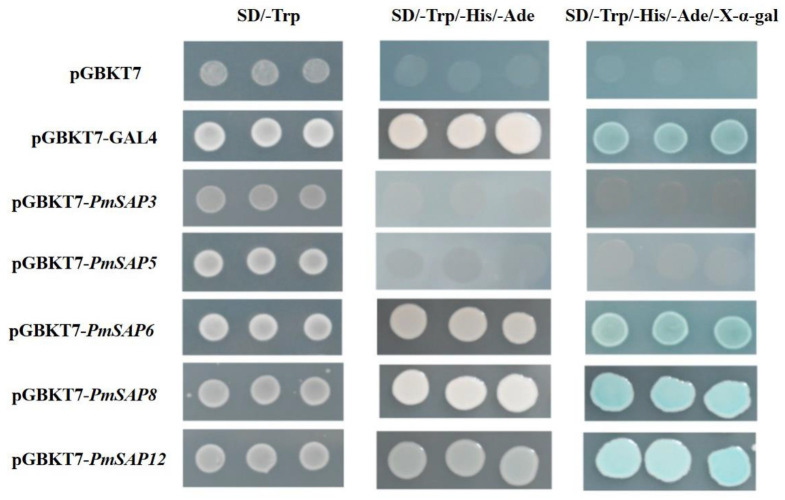
Analysis of transcriptional activity of five *PmSAP* genes. pGBKT7: negative control; pGBKT7-GAL4: positive control.

Furthermore, we conducted transcriptional activity assays for the five genes, namely, *PmSAP3*, *PmSAP5*, *PmSAP6*, *PmSAP8*, and *PmSAP12* (Figure 7). Yeast cells carrying the pGBKT7-*PmSAP3* and pGBKT7-*PmSAP5* fusion expression vectors failed to grow on selective SD/-Trp/-His/-Ade medium. Nevertheless, yeast cells carrying the pGBKT7-*PmSAP6*, pGBKT7-*PmSAP8*, and pGBKT7-*PmSAP12* fusion expression vectors could grow on selective SD/-Trp/-His/-Ade medium. Moreover, upon the addition of X-α-gall, blue spots were displayed on the SD/-Trp/-His/-Ade medium. The research results confirmed that *PmSAP3* and *PmSAP5* function as transcriptional suppressors, while *PmSAP6*, *PmSAP8*, and *PmSAP12* were transcriptional activators. This study provides fundamental insights into their regulatory roles and forms a basis for subsequent research into gene regulation mechanisms.

## 3. Discussion

Proteins featuring A20/AN1 zinc finger domains serve essential functions in modulating animal immunity and plant stress tolerance. To date, the SAP gene family has been identified in multiple plant species, exhibiting considerable variation in gene family size. For instance, 27 SAP genes have been reported in soybean [28], 57 SAP genes in *Brassica napus* [27], 16 SAP genes in *Artemisia annua* [39], 30 SAP genes in *Malus domestica* [18], 17 SAP genes in *Medicago truncatula* [26], 37 SAP genes in *Gossypium hirsutum* [25], 13 SAP genes in *Solanum lycopersicum* [24], 18 SAP genes in *Oryza sativa*, and 14 SAP genes in *Arabidopsis thaliana* [23]. However, reports on the SAP gene family related to stress in *P. massoniana* are not common. This investigation identified 17 SAP gene members in *P. massoniana* (Table S1). These findings significantly enhance our knowledge of the SAP gene family in conifers.

Most of PmSAP proteins vary greatly in structure and exhibit high complexity. The SAP protein sequences of *P. massoniana* are also diverse, ranging from 63 to 334 amino acids in length. Such variation could result from either gene duplications or chromosomal rearrangements [40]. Phylogenetic analysis (Figure 1) shows that all *Arabidopsis* subfamilies have at least one PmSAP protein, indicating that these subfamilies have been conserved throughout long-term evolution without loss. Members of the same subfamily share a genetic structure, while those from different subfamilies display distinct biological activities. For example, most of PmSAP proteins (nine out of seventeen) contain both A20 and AN1 zinc finger domains; by comparison, the other SAPs contain only one or two AN1 zinc finger domains. Nevertheless, some plant species possess SAP proteins containing only a single A20 domain. For instance, among the BnaSAP proteins, seven possess solely an A20 zinc finger domain [27], while *MdSAP22* and *GmSAP23* have only one A20 domain each in apples and soybeans [18,28].

Phylogenetic analysis of SAP proteins from *P. massoniana* and three other plant species revealed that these SAP proteins could be classified into five clades. Most of the proteins within the same phylogenetic clades typically exhibited the same AN1/A20 domain arrangement. Based on the evolutionary relationship, we further investigated the distribution of conserved motifs of SAP proteins in *P. massoniana*. Comprehensive analysis of the 17 *PmSAP* transcription factor family members identified 10 conserved motifs (designated as Motif 1 through Motif 10). Among them, Motifs 1, 2, and 3 are characteristic domains of the SAP family, indicating that these motifs may serve core functional roles in protein functions. Structural domain analysis confirmed that all PmSAP proteins contain either AN1 or A20 zinc finger domains, demonstrating the structural integrity of this gene family (Figure 2). Based on software prediction and verified by tobacco transient transformation experiments, it was found that *PmSAP* is a nuclear localized protein, indicating that SAP TFs may play regulatory functions in the nucleus, aligning with Anqi Su’s findings on SAP gene localization (Figure 4) [41].

Accumulating evidence demonstrates that SAP genes participate in abiotic stress responses across diverse plant species. Under abiotic stress conditions, SAP genes exhibit distinct expression responses. For example, in *Arabidopsis thaliana* (*AtSAP13*) and *Oryza sativa* (*OsSAP7*), some SAP genes exhibit upregulation and are enhanced during early drought stress [16,17], similar to the expression pattern of *PmSAP3/5/7/8* in our study. In contrast, certain SAP family members (such as *AtSAP9*) function as negative regulators of stress responses [19], similar to the continuous downregulation of *PmSAP12*, indicating that the *PmSAP* family members play differential regulatory roles during drought stress (Figure 3). As a coniferous species, the SAP genes of *P. massoniana* may have evolved unique drought response patterns, such as specific expression dependent on needles (photosynthetic tissues), because most *PmSAP* TFs show the highest expression in needles (Figure 5). In addition, *PmSAP* family members display significantly divergent responses to various stress treatments. Among them, *PmSAP8* and *PmSAP12* respond strongly to ABA, MeJA, and H_2_O_2_ treatments; *PmSAP3* and *PmSAP5* show significant upregulation under ETH and NaCl stress (Figure 6). These distinct expression characteristics suggest that different *PmSAP* genes may perform specialized regulatory functions in plant responses to multiple abiotic stresses, providing crucial insights for elucidating their molecular mechanisms. In addition, the transcriptional activation activity in connection with the ability of SAP genes to activate downstream reporter gene expression and the determination of transcriptional activity indicates that *PmSAP6*, *PmSAP8*, and *PmSAP12* are transcriptional activators. *PmSAP3* and *PmSAP5* are transcriptional suppressors (Figure 7), and these results provide a basis for future exploration of gene regulatory networks.

In summary, *PmSAP*s exhibit conserved structural and functional characteristics similar to SAP genes in other plant species [21], and they serve as key regulators in *P. massoniana* under abiotic stress conditions. These results substantially advance our comprehension of the stress responses mediated by SAPs in *P. massoniana* and provide a theoretical foundation for subsequent functional gene research.

Based on this, subsequent research should give priority to the following aspects: (1) functional validation of key *PmSAP* genes (such as *PmSAP3/5/8/12*) through transgenic approaches in *P. massoniana* and (2) elucidation of their upstream regulatory networks and downstream target genes. These efforts may ultimately contribute to the genetic improvement of stress resistance in *P. massoniana* and related forest species.

## 4. Materials and Methods

### 4.1. Identification of the SAP Genes in P. massoniana

The SAP protein domains (PF01428 and PF01754) were initially retrieved from the Pfam database (https://pfam.xfam.org/, accessed on 14 December 2023) to construct a Hidden Markov Model (HMM). Using the HMM profile, we systematically searched for SAP proteins across four *P. massoniana* transcriptomes: drought stress transcriptome (PRJNA595650) [42], CO_2_ stress transcriptome (PRJNA561037) [43], tender shoots transcriptome (PRJNA655997), and *P. massoniana* inoculated with the pine wood nematode transcriptome (SRA: PRJNA66087) [44]. For additional confirmation of sequence reliability, the candidate SAP protein domains were predicted by CD-Sear in NCBI (https://www.ncbi.nlm.nih.gov/Structure/cdd/wrpsb.cgi, accessed on 5 January 2024), and the predicted SAP domains were compared. Proteins with similarity exceeding 97% in the transcriptome data were deleted, and proteins with incomplete domains were also removed. Thus, protein sequences that met the criteria were selected (Table S1).

### 4.2. Bioinformatics and Phylogenetic Analysis of PmSAP Proteins

The physicochemical properties of the SAP protein of *P. massoniana*, including molecular weight (MW), isoelectric point (pI), aliphatic amino acid index, instability index, and hydrophilicity index, were analyzed using the ExPASy website (https://web.expasy.org/protparam/, accessed on 11 January 2024). The SAP protein sequences of *Arabidopsis thaliana*, *Pinus tabuliformis*, and *Oryza sativa* were obtained from the Plant Transcription Factor Database (https://planttfdb.gao-lab.org/, accessed on 19 January 2024), and a phylogenetic tree was generated using MEGA7, employing the neighbor-joining (NJ) algorithm with 1000 bootstrap replicates [45]. The phylogenetic tree was then edited using the EvolView online tool (https://www.evolgenius.info/evolview, accessed on 22 January 2024). The distribution of conserved motifs of the *PmSAP* protein was analyzed through the MEME online website (https://meme-suite.org/meme/, accessed on 5 February 2024), which identified 10 conserved motifs. TBtools software (version 2.067) was utilized for both visualization and analytical processing of the results.

### 4.3. RNA-Seq Data Analysis Under Drought Stress

RNA sequencing (RNA-seq) data from drought stress transcriptome (PRJNA595650) were utilized to investigate the expression patterns of *PmSAP* genes. Using the FPKM (fragments per kilobase of exon per million fragments mapped), we evaluated transcript abundance for *PmSAP* genes. Gene expression patterns were visualized as partial heatmaps in TBtools (2.067), based on the log_2_ (FPKM + 1) values, and analyzed on the row scale [46].

### 4.4. Subcellular Localization of PmSAP Proteins

We employed the Cell-PLoc online tool (http://www.csbio.sjtu.edu.cn/bioinf/Cell-PLoc/, accessed on 28 February 2024) for preliminary subcellular localization analysis of PmSAP proteins. Then, transient transformation experiments were conducted on tobacco leaves. The open reading frames (ORFs) of *PmSAP3*, *PmSAP5*, *PmSAP6*, *PmSAP8*, and *PmSAP12* were obtained through gene cloning. For gene cloning and vector construction, the required primer sequences are provided in the Supplementary Materials (Table S5). The ORF regions (excluding termination codon) were ligated with the pCAMBIA1302-eGFP vector to construct the 35S::*PmSAP*-eGFP expression vector. The vectors were transformed into the Agrobacterium GV3101, and the RNA silencing inhibitor P19 was co-cultured with it at 28 °C for 36 h [47]. Then, the cells were resuspended in the buffer (10 mM MES, 200 μM acetosyringone, and 10 mM MgCl_2_) and combined with P19 in a 1:1 proportion to prepare the infiltration solution. The resulting infiltration solution was injected into 30-day-old *Nicotiana benthamiana* leaves. After 48 h of dark cultivation, using the LSM710 confocal laser scanning microscope, GFP fluorescence was detected at 488 nm excitation, and DAPI signals were captured at 405 nm excitation.

### 4.5. Plant Materials and Abiotic Stress Treatments

The plant materials consisted of one-year-old *P. massoniana* seedlings obtained from the State Key Laboratory of Tree Genetics and Breeding at Nanjing Forestry University. Five distinct tissue types were collected from healthy, uniformly grown seedlings: terminal bud (TB), stem (S), needle (N), root (R), and phloem (P). Eight abiotic stress treatments were applied as follows. The treatments were carried out by spraying 100 μM abscisic acid (ABA), 10 mM methyl jasmonate (MeJA), 1 mM salicylic acid (SA), 50 μM ethylene (ETH), and 10 mM H_2_O_2_ on the needles of *P. massoniana* [48,49]. Osmotic stress was imposed by irrigating the soil with 200 mM NaCl solution; drought stress was established through natural evaporation over a 20-day period post-initial watering (day 0); and mechanical injury was induced by cutting the upper half of the needles. Samples were taken from the needles at 0 d, 3 d, 7 d, 12 d, and 20 d under drought stress (0 d as control). Samples at 0 h, 3 h, 6 h, 12 h, and 24 h were taken for other stresses after treatment (0 h as control) [50]. Three biological replicates were maintained for all treatments.

### 4.6. RNA Extraction and qRT-PCR Analysis

The FastPure Universal Plant Total RNA Isolation Kit (RC411-01, Vazyme Biotech, Nanjing, China) was employed to extract RNA from *P. massoniana* needle samples. The integrity of RNA samples was checked by electrophoresis on a 1% agarose gel, followed by concentration measurement. The first strand cDNA synthesis kit (Yeasen Biotechnology, Shanghai, China) was used to synthesize cDNA from 1 µg of total RNA. The StepOne Plus real-time PCR System (Foster City Applied Biosystems, Foster City, CA, USA) was employed for qRT-PCR, with *PmTUA* serving as the internal reference gene [51]. Each PCR mixture was 10 μL, consisting of 1 μL of 20-fold diluted cDNA, 5 μL of SYBR Green Master Mix (Yeasen Biotechnology, Shanghai, China), 0.4 μL of forward and reverse primers (10 μM), and 3.2 μL of ddH_2_O. All primer sequences are provided in the Supplementary Materials (Table S6). The amplification conditions were 95 °C for 2 min pre-denaturation, 95 °C for 10 s denaturation, 60 °C for 30 s extension, and a total of 40 cycles. Three independent technical replicates were conducted for every reaction. Gene expression level of *PmSAP* was determined through the 2^−∆∆CT^ method, with subsequent statistical analysis conducted in GraphPad Prism 8.0 software.

### 4.7. Transcriptional Activation Assay

To determine whether PmSAP proteins possess transcriptional activation activity, we constructed recombinant pGBKT7-*PmSAP* vectors containing the complete open reading frames (ORFs) of *PmSAP3*, *PmSAP5*, *PmSAP6*, *PmSAP8*, and *PmSAP12*. The primer sequences used for vector construction are provided in the Supplementary Materials (Table S5). These fusion vectors, along with the empty pGBKT7 vector as a negative control, were subsequently transformed into the yeast strain AH109 (YC1010, Weidi Biotech, Shanghai, China). Following incubation at 28 °C for 3 days on medium plates lacking tryptophan (SD/-Trp), yeast single colonies were collected and subjected to PCR identification with 10 μL ddH_2_O. After positive detection, the remaining positive bacterial liquid was diluted to 200 μL with ddH_2_O. Use a pipette to draw 5 μL of the diluted solution and transfer it onto three different yeast growth media: lacking tryptophan (SD/-Trp), lacking tryptophan/histidine/adenine (SD/-Trp/-His/-Ade), and SD/-Trp/-His/-Ade medium supplemented with X-α-gal. Finally, the yeast cell growth was monitored and documented.

## 5. Conclusions

This research presents the first systematic identification and classification of *PmSAP* TFs in *P. massoniana*. We identified 17 *PmSAP* genes, which phylogenetic analysis classified into five distinct clades, and 10 conserved motifs were identified. Bioinformatics prediction and subcellular localization confirmed the nuclear localization of PmSAP proteins. Meanwhile, transcription auto-activation tests showed that *PmSAP6/8/12* are transcriptional activators, while *PmSAP3* and *PmSAP5* are suppressors. Notably, the response of *PmSAP* family members to different stress treatments shows significant differences. For instance, *PmSAP8* and *PmSAP12* respond strongly to ABA, MeJA, and H_2_O_2_ treatments, while *PmSAP3* and *PmSAP5* show significant upregulation under ETH and NaCl stress. This research reveals previously unreported SAP gene expression in *P. massoniana*, establishing a crucial foundation for subsequent research on the stress response mechanism mediated by *PmSAP*.

## Data Availability

All supporting data have been deposited in the Supplementary Materials.

## Supplementary Materials

The following supporting information can be downloaded at https://www.mdpi.com/article/10.3390/plants14111592/s1, Table S1: Sequences of the SAP family proteins from *P. massoniana*; Table S2: The physicochemical properties of SAP TF family members in *P. massoniana*; Table S3: Motif sequences of the *PmSAP* TFs; Table S4: Subcellular localization prediction of *PmSAP*s of *P. massoniana;* Table S5: The sequences of primers used in different experiments.
